# Deciphering the Growth Behaviour of *Mycobacterium africanum*

**DOI:** 10.1371/journal.pntd.0002220

**Published:** 2013-05-16

**Authors:** Florian Gehre, Jacob Otu, Kathryn DeRiemer, Paola Florez de Sessions, Martin L. Hibberd, Wim Mulders, Tumani Corrah, Bouke C. de Jong, Martin Antonio

**Affiliations:** 1 Institute of Tropical Medicine, Antwerp, Belgium; 2 Medical Research Council Unit, Fajara, The Gambia; 3 University of California, Davis, Davis, California, United States of America; 4 Genome Institute of Singapore, Singapore, Singapore; 5 New York University, New York, New York, United States of America; University of Tennessee, United States of America

## Abstract

**Background:**

Human tuberculosis (TB) in West Africa is not only caused by *M. tuberculosis* but also by bacteria of the two lineages of *M. africanum*. For instance, in The Gambia, 40% of TB is due to infections with *M. africanum* West African 2. This bacterial lineage is associated with HIV infection, reduced ESAT-6 immunogenicity and slower progression to active disease. Although these characteristics suggest an attenuated phenotype of *M. africanum*, no underlying mechanism has been described. From the first descriptions of *M. africanum* in the literature in 1969, the time to a positive culture of *M. africanum* on solid medium was known to be longer than the time to a positive culture of *M. tuberculosis*. However, the delayed growth of *M. africanum*, which may correlate with the less virulent phenotype in the human host, has not previously been studied in detail.

**Methodology/Principal Findings:**

We compared the growth rates of *M. tuberculosis* and *M. africanum* isolates from The Gambia in two liquid culture systems. *M. africanum* grows significantly slower than *M. tuberculosis*, not only when grown directly from sputa, but also in growth experiments under defined laboratory conditions. We also sequenced four *M. africanum* isolates and compared their whole genomes with the published *M. tuberculosis* H37Rv genome. *M. africanum* strains have several non-synonymous SNPs or frameshift mutations in genes that were previously associated with growth-attenuation. *M. africanum* strains also have a higher mutation frequency in genes crucial for transport of sulphur, ions and lipids/fatty acids across the cell membrane into the bacterial cell. Surprisingly, 5 of 7 operons, recently described as essential for intracellular survival of H37Rv in the host macrophage, showed at least one non-synonymously mutated gene in *M. africanum*.

**Conclusions/Significance:**

The altered growth behaviour of *M. africanum* might indicate a different survival strategy within host cells.

## Author Summary

*Mycobacterium tuberculosis* and *Mycobacterium africanum* are the two major lineages within the *M. tuberculosis* complex that cause human tuberculosis in West Africa. Despite being closely related, the outcome after infection differs between these two pathogens. Although *M. africanum* has not yet been studied to the same extent as *M. tuberculosis*, *M. africanum* is less likely to stimulate the host immune system or to progress to active disease. We hypothesized that this somewhat attenuated phenotype is due to the slower growth of *M. africanum* within the host. Therefore, we analysed clinical isolates from 522 patients with tuberculosis in The Gambia. *M. africanum* West Africa 2 strains grew more slowly than *M. tuberculosis*. We sequenced four *M. africanum* strains and identified several candidate genes that may cause the growth-attenuation of the bacteria. Describing the fundamental genomic and phenotypic differences between *M. tuberculosis* and *M. africanum* will enable us to better understand the virulence mechanisms that make *M. tuberculosis* one of the most successful bacterial pathogens, and to discover potential strategies to interfere with mycobacterial pathogenicity.

## Introduction

*Mycobacterium africanum*, a member of the *Mycobacterium tuberculosis* complex, was first described in 1968 in Dakar, Senegal [1]. Infections with *M. africanum* are generally geographically restricted to human populations in West Africa, and are not well understood [2]. Molecular techniques have since refined the classification of the sub-species *M. africanum* into *M. africanum* West African 1, common around the Gulf of Guinea, and *M. africanum* West African 2, mainly found in Western West Africa [3], [4]. Although up to 30–40% of all human tuberculosis in West Africa is caused by either of the two *M. africanum* lineages [2], basic research on these clinically important mycobacteria was neglected to date. However, an improved understanding of the biology of this mycobacterial lineage will also give clues about genetic functions in the closely related *M. tuberculosis*.

The biochemical characteristics of *M. africanum* vary; at times, they resemble those of *M. bovis*, and, at times, *M. tuberculosis* [5]. Clinically and epidemiologically, *M. africanum* behaves very differently from *M. tuberculosis*. For instance, studies from The Gambia showed that *M. africanum* West African 2 is associated with HIV infection [2], reduced ESAT-6 immunogenicity [6] and a slower progression to active disease [7]. These features suggest an overall attenuation of the bacterium, yet no underlying mechanism has been identified to date. From the first descriptions of *M. africanum*, the time to detection on solid medium was known to be longer for *M. africanum* compared to *M. tuberculosis*. This delayed growth, which may explain the reduced virulence of *M. africanum*, has not previously been investigated.

We determined the bacterial growth rate of molecularly characterized lineages of the *M. tuberculosis* complex collected from The Gambia in two liquid culture systems, both directly from sputum and in carefully controlled growth experiments. *M. africanum* West-African 2 (from now on referred to as *M. africanum*) grows significantly slower than *M. tuberculosis* in all of the culture systems we used. By comparisons of genetic sequence data, *M. africanum* strains have several mutations in genes that were previously associated with growth-attenuation in *M. tuberculosis* H37Rv. This high mutation frequency was also observed in functional groups of molecular membrane transport systems that translocate macromolecules and nutrients across the cell membrane into the bacterial cell. We conclude that the altered growth behaviour of *M. africanum* may be a different survival strategy within the host.

## Materials and Methods

### Mycobacterial growth curves from sputum and from standardized inoculum

In the context of several TB cohort studies, we collected clinical isolates from patients with smear positive pulmonary TB. Each TB patient submitted up to three sputum samples. Sputum was decontaminated using NALC-NaOH and inoculated into either BACTEC MYCO/F-Sputa vials (for the BACTEC 9000, BD) and/or BACTEC MGIT 960 Tubes supplemented with PANTA (for BACTEC MGIT 960, BD). The tubes were incubated at 37°C and the “Time to Positivity” (manufacturer-set threshold: 75 Growth Units) was recorded in days. Tubes were incubated for a maximum of 42 days.

In a second experiment we compared the growth rates of *M. tuberculosis* laboratory strain Mt14323 [8] and clinical *M. africanum* isolate ITM 080552 in a controlled laboratory setting, using defined inocula. For each strain, a standardized inoculum was prepared from a fresh subculture of 21 days with a turbidity of McFarland N° 0.5. The OD_492 nm_ and OD_595 nm_ were measured and the bacterial suspension was adjusted to OD = 0.01–0.03. A dilution of 1∶10 was prepared in distilled water. From this dilution we made a half logarithmic dilution series and 100 µl of each dilution was inoculated in triplicate into MGIT960. To estimate colony-forming-units (CFU) each inoculum was plated on 7H11 plates. Growth curves were monitored using the BD Epicentre software, data were extracted, and the length of the lag phase or “Time to Positivity” for each strain to reach the “positivity threshold” of 75 growth units (GU) was measured. Furthermore, the actual growth rate or doubling time was determined as the time needed for a strain to grow from 5000GU to 10000GU. We used the non-parametric Wilcoxon rank sum test to compare the median time to positivity for *M. tuberculosis* versus *M. africanum*.

### Ethical statement

The samples used are all from the MRC strain collection, which comprises strains from various studies that were conducted over the last years. All these studies obtained ethical approval, informed consent from patients and samples were anonymized.

### Genotyping mycobacterial isolates

Genotyping was done using spoligotype analysis [9] and PCR for Large Sequence Polymorphisms [4] on the mycobacterial DNA from one isolate from each patient, with the assumption that all samples from the same patient contained the same mycobacterial isolate. Isolates were grouped in phylogenetically distinct lineages within the *M. tuberculosis* complex, as previously defined [4].

### Next generation sequencing for whole bacterial genomes

We sequenced the genomes of four *M. africanum* West Africa 2 isolates, three that originated from The Gambia and one publicly available strain from Senegal. We re-sequenced the published strain GM041982 [10], two randomly selected Gambian isolates 03/03910 and 03/030671 from MRC's strain collection, and strain ATCC 35711.

#### Library preparation

2.5 µg of genomic DNA products of each sample were combined and fragmented into a peak size range of 200–400 bp using the Covaris S2 (Covaris, Woburn, MA, USA) (shearing conditions - Duty cycle: 20%; Intensity: 4; Cycles per burst: 200; Time: 360 seconds). After fragmentation, the samples were purified using the Qiagen PCR purification kit (Qiagen, Valencia, CA, USA). Fragmented products were quality-checked (2100 Bioanalyzer on a DNA 1000 Chip, Agilent Technologies, Santa Clara, CA, USA). The NEB Next DNA Sample Prep Master Mix kit (New England Biolabs, Ipswich, MA, USA) was used. Library preparation entailed: end-repair, A-tailing and ligation of adapters according to the manufacturer's instructions. Size selection was conducted on a Pipen Prep from Sage Science, fragments in the range 300–500 bp were selected. Then a quality-check of the size selected product was run on the 2100 Bioanalyzer (DNA High Sensitivity DNA Chip). Finally, using the Multiplexing Sample Preparation Oligonucleotide Kit (Illumina, San Diego, CA, USA), samples underwent 14 PCR cycles to incorporate indexes followed by Agencourt AMPure XP magnetic bead (Beckman Coulter, Brea, CA, USA) clean up according to manufacturer's instructions. One more quality check was conducted using the Bioanalyzer with a run on a DNA 1000 Chip, and all samples were adjusted to a final concentration of 10 nM. A qPCR step was performed to ensure all material sent for sequencing contained the adaptors and indexes. We used the LightCycler 480 SYBR Green I Master mix (Roche Applied Science, Indianapolis, IN, USA) in a LightCycler 480 II real time thermal cycler (Roche Applied Science, Indianapolis, IN, USA) according to the manufacturer's instructions.

#### Multiplexed sequencing

Next generation sequencing was done using Illumina Hiseq 2000 flow cell, 2×76 base pair-end runs. PhiX was used as control. Sequencing was carried out by Genome Technology Biology team in Genome Institute of Singapore.

#### Analysis of whole genome sequences

Unix Korn Shell was used to access the server and perform the analysis. Scripts written by the bioinformatics team in GIS were used to do assembly, call SNPs and build a phylogeny tree for the sample set. CLC Genomics was used to visualize quality of the reads, mapped SNPs and align sequences.

### Genetic comparison of *M. tuberculosis* H37Rv and *M. africanum*

We compared selected genes from the *M. tuberculosis* H37Rv genome [11] with their respective homologues of the sequenced *M. africanum* strains. We only considered single nucleotide polymorphisms (SNPs) or deletion/insertion polymorphism (DIPs) that were common to all four *M. africanum* strains. Genes that were only mutated in some of the sequenced strains were considered to be uncommon to *M. africanum* and were considered wildtype genes. The analysed set of genes which is responsible for attenuated growth *in vitro* was extracted from a previous publication [12]. Genes and operons essential for *in vivo* growth within macrophages were recently published [13]. Genes encoding nutrient and macromolecule transport mechanisms were identified from the literature [14]–[17] and by a NCBI PubMed search. Additionally, genes annotated as transporters were identified in the TubercuList database (http://tuberculist.epfl.ch/). In both searches, genes encoding transport proteins with unknown substrate specificity or annotated drug/antibiotic efflux pumps were excluded from the analysis. To compare the proportion of genes carrying non-synonymous SNPs between groups the Fisher's exact test was conducted and the results were considered significant at the level of p≤0.05, assuming the likelihood of a Type I error was α = 0.05.

### Predicting the impact of non-synonymous mutations on protein function

To understand whether an amino acid substitution affected protein function in *M. africanum*, we conducted an analysis using the SIFT (“Sorting Intolerant from Tolerant”) Sequence algorithm (http://sift.bii.a-star.edu.sg/www/SIFT_seq_submit2.html) [18]. The parameters were used at their default setting and the gene sequences and respective substitutions were analysed using the “UniProt-SwissProt+TrEMBL 2010_09 database” as reference.

## Results

### Growth curves from sputum

**Figure 1:**
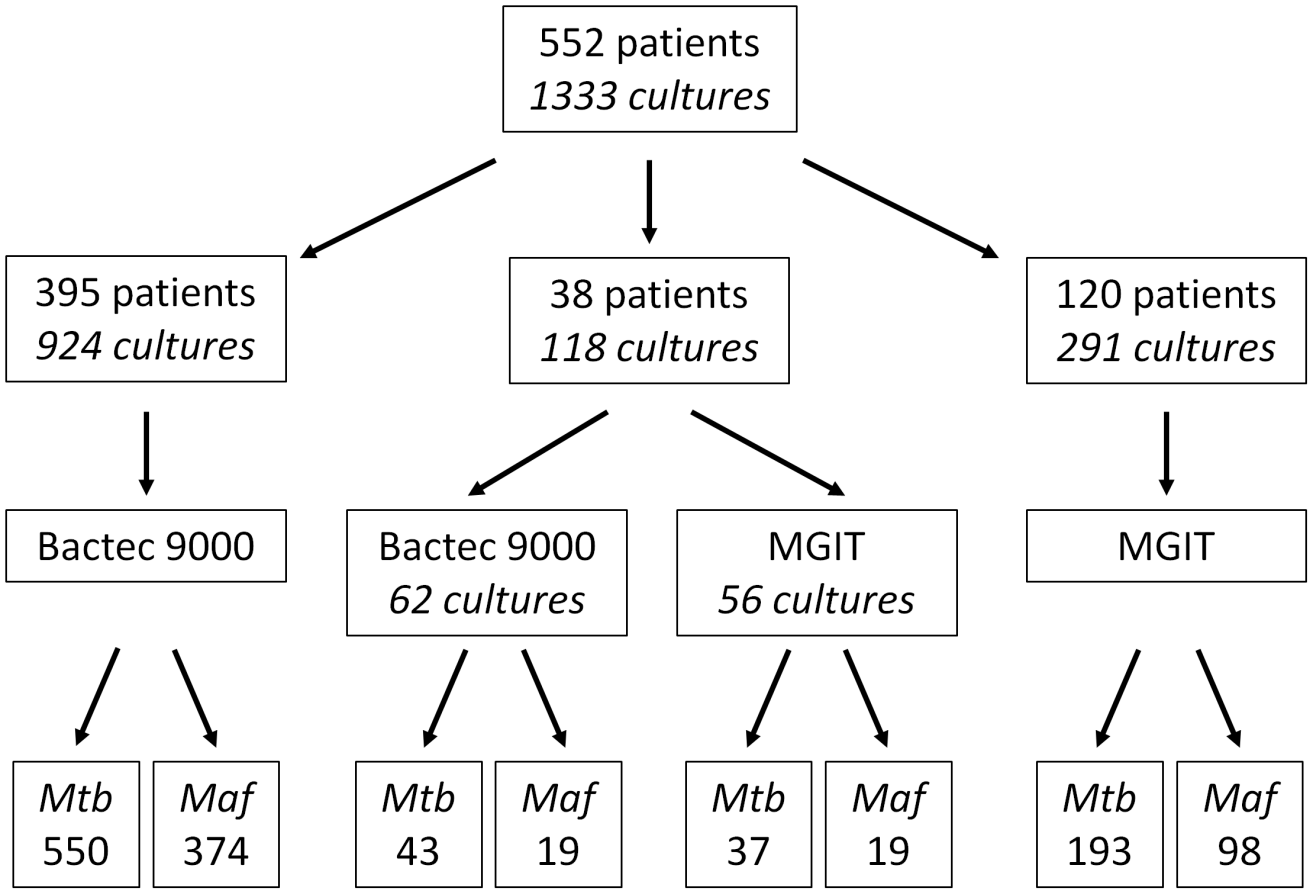
Culture methods and spoligotyping results from isolates obtained from sputum samples of 552 patients in the study.

**Figure 2:**
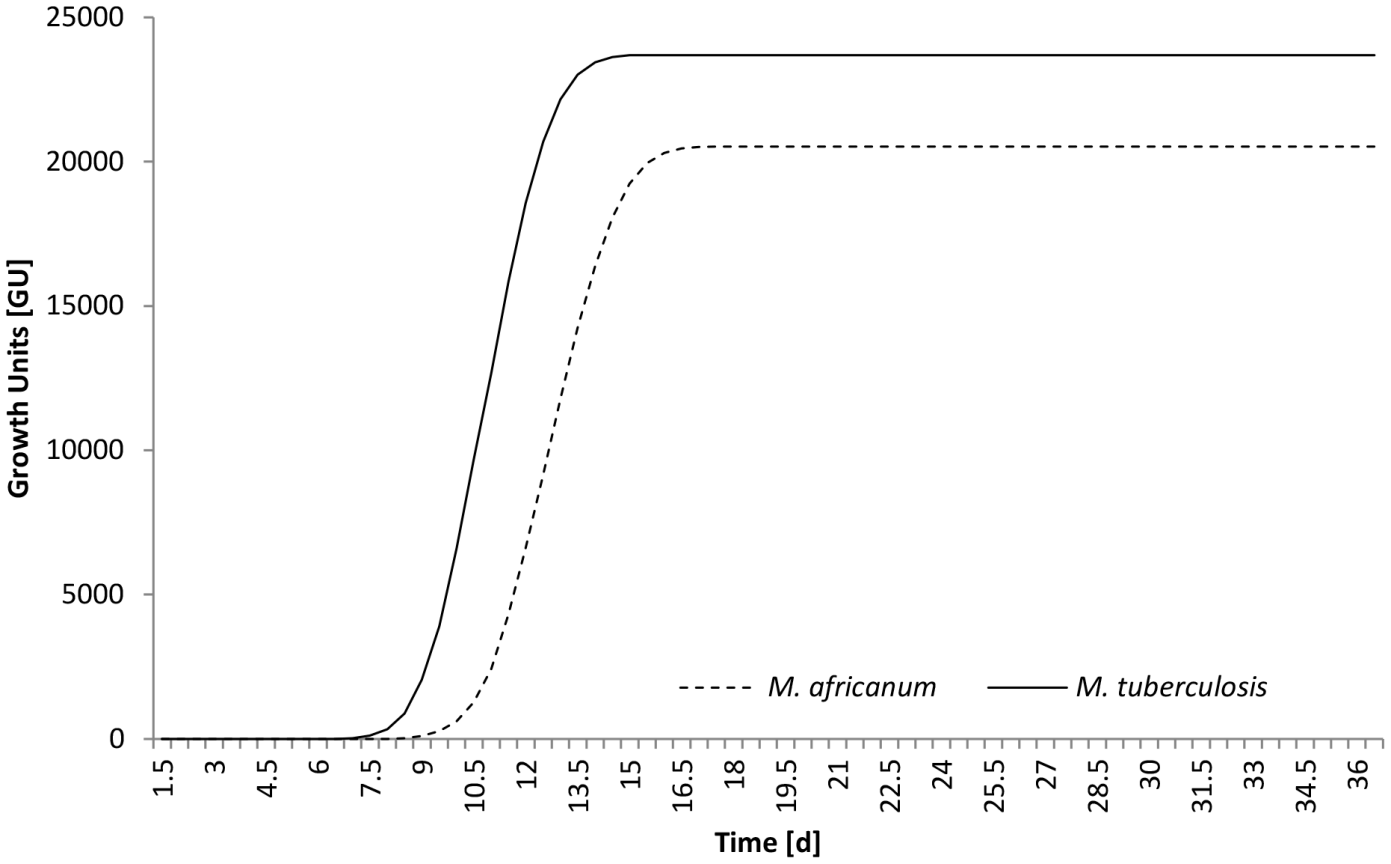
Frequency distributions for the Time-to-Positivity of mycobacterial cultures in two liquid culture systems. All samples were incubated for 42 days. Upper panels: results for the Bactec 9000, Lower panels: Bactec MGIT 960, solid bars: *M. tuberculosis*, open bars: *M. africanum*.

Among isolates from 552 TB cases the prevalence of *M. africanum* West Africa 2 (n = 223) was 40%, consistent with previously reported results [19]. From these 552 patients a total of 1333 positive cultures (*M. tuberculosis* n = 823, *M. africanum* n = 510) were obtained and analysed in the study (Figure 1). In both liquid culture systems, the median time to culture positivity was significantly shorter for *M. tuberculosis* (Bactec 9000: 13d, MGIT960: 9d) relative to *M. africanum* (Bactec 9000: 21d, MGIT960: 15d) (see Table 1 and Fig. 2).

**Table 1:** Growth of *M. tuberculosis* complex isolates from sputum in The Gambia in two liquid culture systems.

	Bactec 9000	Bactec MGIT
***M. africanum***		
Number of cultures	393	117
Median days to growth	21	15
Range	2–42	2–40
***M. tuberculosis***		
Number of cultures	593	230
Median days to growth	13	9
Range	2–42	1–22
P value*	<0.00005	<0.00005

* Wilcoxon rank sum test comparing median time for *M. africanum* vs. *M. tuberculosis*.

### Growth curve from standardized inoculum

**Figure 3:**
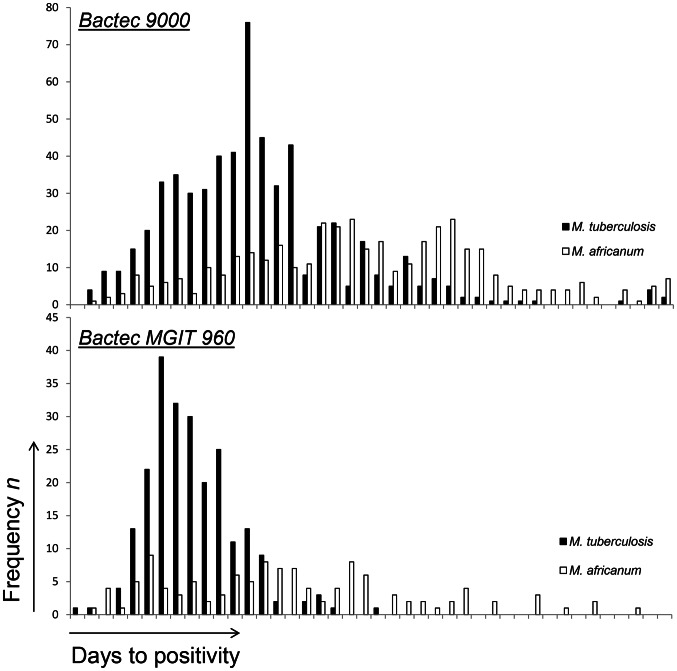
*In vitro* growth curves from standardized inocula. *M. tuberculosis* (solid line) and *M. africanum* (dashed line) were grown from standardized inocula in Bactec MGIT 960 and measured growth units (GU) are plotted versus time [days].

To further compare the growth dynamics of the two lineages, we inoculated 5.9×10^3^ CFU/ml and 8.3×10^3^ CFU/ml of *M. tuberculosis* and *M. africanum*, respectively, into MGIT tubes and incubated them at 37°C. The lag phase or “time to positivity” was 175.2 hours (7.30 days) for *M. tuberculosis* strain Mt14323 and 213.00 hours (8.88 days) for *M. africanum* strain ITM 080552. Furthermore, we determined the doubling time of *M. tuberculosis* to be 20.16 h, in contrast to the doubling time of 24.12 h *for M. africanum* (see Figure 3).

### Genetic comparison of *M. tuberculosis* H37Rv and *M. africanum*

#### Growth attenuating genes

Based on transposon insertion and gene-disruption, a set of 42 growth-attenuating genes was previously identified [12]. Each of these genes resulted in growth-attenuation when inactivated through transposon insertion. In our study, 12 of the 42 growth-attenuating genes had non-synonymous mutations, and 4 of these genes were affected in their protein function as predicted by SIFT analysis. For a detailed list of the genes and their mutations see table 2.

**Table 2:** List of growth-attenuating genes with non-synonymous or frameshift mutations in *M. africanum*.

Gene name H37Rv	MAF homologue	Product/Function	Mutation in MAF	Amino acid change in MAF	SIFT analysis
Rv0862c	MAF_08710	Conserved protein/Function unknown	C480A, T2246C	None, L749V	Affects protein function
Rv1096	MAF_11110	Possible Glycosyl Hydrolase/Probably involved in Carbohydrate degradation	C814T	P272S	-
Rv1125	MAF_11410	Conserved hypothetical protein/Function unknown	A301G, G855C, T897C	S101G	-
Rv1178	MAF_11970	Probable Aminotransferase/Function unknown	C741T, C953T	A318V	-
*glgP*	*glgP*	Probable Glycogen phosphorylase/Allosteric enzyme in carbohydrate metabolism	G2192A, G2401T	G731D, A801S	-
Rv1592c	MAF_16040	Conserved hypothetical protein/Function unknown	A963G, A964G	I322V	-
Rv2112c	MAF_21240	Deamidase/Deamidates the C-terminal Glutamine of PUP	C20G, C1499T	P7R, A500V	Affects protein function (low confidence)
*aceE*	*aceE*	Pyruvate Dehydrogenase E1 component/involved in energy metabolism	G2329A	A777T	Affects protein function
*mbtB*	*mbtB*	Phenyloxazolin synthase/Biogenesis of the Hydroxyphenyl-oxazoline-containing siderophore mycobactins	C2932T	L978V	-
*recA*	*recA*	Recombinase A/Nucleotide Excision Repair	A1697C	Q566P	Affects protein function
Rv3282	MAF_32920	Conserved hypothetical protein/Function unknown	C434A	T145K	-
glpK	glpK	Probable Glycerol Kinase/Glycerol utilization	T1379C, (Pseudo-gene in GM041182)	V460A, (Pseudo-gene in GM041182)	(Pseudogene in GM041182)

#### Essential genes

*M. tuberculosis* genes were previously classified into two groups: essential or non-essential for *in vitro* growth [12].We selected the 614 essential genes described for *M. tuberculosis* H37Rv and genetically compared them with their respective homologues in *M. africanum*. We found that 132 (21%) of 614 essential *M. africanum* genes contained non-synonymous mutation(s) which resulted in an amino acid change within the respective proteins. We considered the 21% percentage difference to be the baseline difference between the genomes of *M. tuberculosis* H37Rv and *M. africanum* (see Table 3).

**Table 3:** Comparison of certain gene groups in *M. africanum* compared to essential genes mapped against *M. tuberculosis* H37Rv.

	Wildtype genes		Genes with non-synonymous/frameshift mutations		Total	OR, (CI95%)	Fisher's exact test (one-tailed p-value)
	N	%	N	%			
***Transporter genes***	89	(67)	43	(33)	132	**0.6 (0.38–0.86)**	**0.0054**
Peptide transport	7	(78)	2	(22)	9	1.0 (0.21–5.08)	0.6582
Amino acid transport	18	(78)	5	(22)	23	1.1 (0.39–2.94)	0.5626
Carbohydrate transport	13	(72)	5	(28)	18	0.7 (0.25–2.03)	0.3475
Nitrogen transport	7	(100)	0	(0)	7	-	0.1632
Phosphorous transport	13	(93)	1	(7)	14	3.9 (0.50–29.88)	0.1377
Sulphur transport	3	(43)	4	(57)	7	**0.2 (0.05–1.01)**	**0.0553**
Lipid transport	6	(43)	8	(57)	14	**0.2 (0.07–0.60)**	**0.0045**
Ion transport	22	(55)	18	(45)	40	**0.3 (0.17–0.64)**	**0.0012**
***Required operons/genes for intracellular survival (from *** [***13***] ***)***	23	(68)	11	(32)	34	0.6 (0.27–1.20)	**0.1044**
***Essential genes for optimal in vitro growth (from *** [***12***] ***)***	482	(79)	132	(21)	614	1	**-**

#### Transporter genes

**Figure 4:**
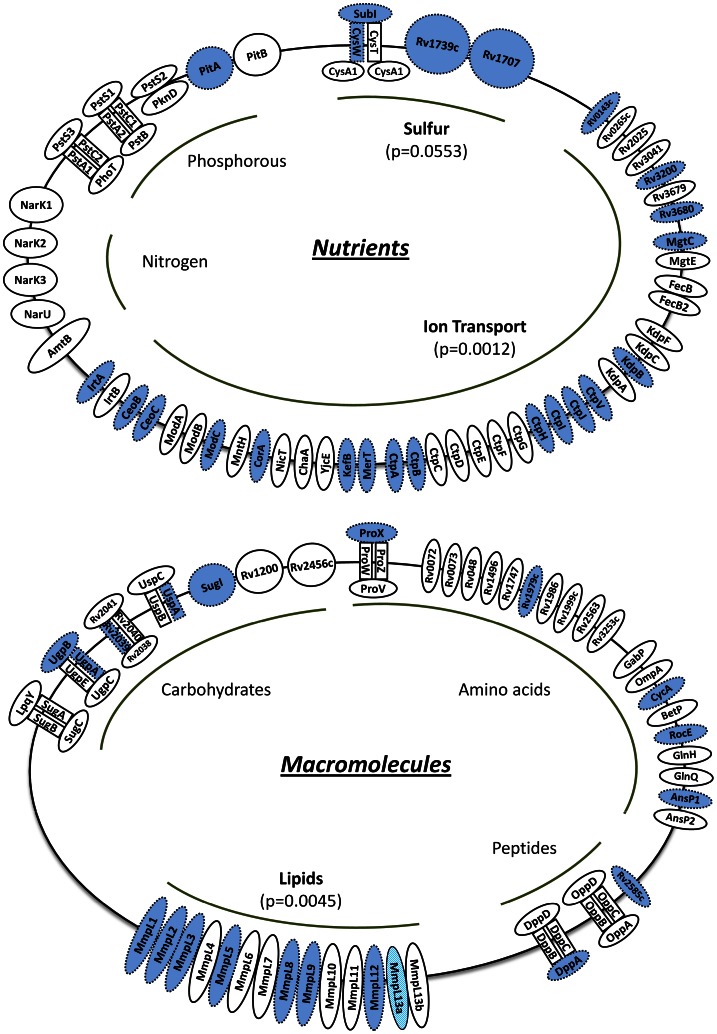
Schematic overview of molecular transport mechanisms present in *M.* africanum. Genetic information of 132 genes encoding various families of membrane transporters was compared between the sequenced *M. africanum* strains and *M. tuberculosis* H37Rv. The analysis comprised both, transport mechanisms specific to nutrients and macromolecules (upper and lower figure). Genes that are identical to the wildtype *M. tuberculosis* H37Rv gene homologue are displayed with a solid border line and white background. Genes, with non-synonymous mutation are displayed in blue with dashed lines. Genes with a frameshift mutation, are displayed with striped background.

We identified a total of 132 genes that were common to both *M. tuberculosis* H37Rv and *M. africanum* and that were described or annotated as genes encoding structural components of membrane transporters from the literature and publicly available databases. The 132 genes included transporters specific for nutrients as well as for the transport of macromolecules. We found that *M. africanum* genes encoding sulphur transporters, ion transporters and fatty acids transporters were significantly more likely to carry a non-synonymous mutation than essential *M. africanum* genes (see Table 3 and Figure 4). No such effect was observed in genes encoding carbohydrate, amino acid or peptide transporters. Interestingly, we found transporter systems specific for nitrogen were hyper- conserved and were less likely to be mutated when compared with essential genes.

#### Genes essential for intracellular survival

**Figure 5:**
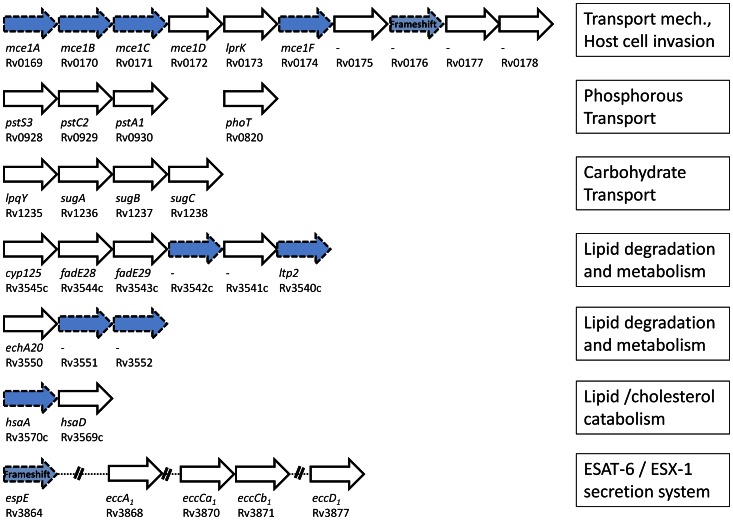
Status of putative *M.* africanum operons essential for intracellular survival. Seven operons were previously defined as essential for growth within the macrophage [13]. Genes that are identical to the wildtype *M. tuberculosis* H37Rv gene homologue are displayed with a solid border line and white background. Genes, with a non-synonymous mutation are displayed in blue with dashed lines. Genes with a frameshift mutation, are displayed with striped background.

We studied a group of 7 operons that were previously described as crucial for the intracellular survival of *M. tuberculosis* H37Rv in macrophages [13]. We found that five out of seven operons had at least one mutated gene in *M. africanum* (see Fig. 5). Although the *pstA1* gene of the phosphate transport operon was mutated in *M. africanum* GM041182 and ATCC35711, this was not a common trait of all analysed *M. africanum* strains.

## Discussion

*M. africanum* grows slower than *M. tuberculosis*, with delayed culture positivity (by 4–6 days) when grown from sputum in modern liquid culture systems. Although these liquid culture systems are only indirectly measuring growth by detecting oxygen or radioactive precursor consumption as a proxy for growth, they are well suited to compare the growth behaviour of different bacterial isolates. The observed growth differences between the two lineages were further emphasized by a survival analysis which was adjusted for smear grade, and we estimated a Hazard ratio (HR) = 0.40 (95%CI 0.35–0.47, p<0.0005). Consistent with this diagnostic observation, we determined a longer doubling time of *M. africanum* in growth experiments in which the inoculum was carefully standardized by CFU. One limitation of this approach is that it is not clear whether cording differs between *M. africanum* and *M. tuberculosis*, which could potentially impact on CFU standardization. Also testing a wider range of isolates together with comparative genomics could enhance the power of the *in vitro* experiments in the future. However, both our findings on growth from sputum and standardized inoculum are consistent with other studies, as the measured doubling times for both of the lineages were in the same range as previously described by Bold *et al.* [20]. Our results also reproduced the initial observations from Senegal in 1968, when isolates, identified as *M. africanum* by biochemical methods, yielded growth on solid media later than *M. tuberculosis* isolates [1]. Therefore, Castets in 1979 recommended incubation for 90 days for the detection of *M. africanum* on solid media [21]. However, with the advent of modern automated liquid culture systems, the original recommendations need to be adjusted and redefined. For the current study, we incubated all samples for 42 days, as recommended by the manufacturer of the liquid culture systems. Although 42 days are sufficient to detect *M. tuberculosis* strains, the frequency distributions indicated that for *M. africanum* strains a longer incubation period might be advisable. For instance, in the Bactec 9000, only seven cultures turned positive on the very last day of the incubation period. For this reason, the Bactec MGIT960 seems preferable and better suited for the cultivation of *M. africanum*, as overall incubation times decreased and detection of culture positivity can be achieved faster. Therefore to evaluate the potential of these liquid cultures systems as diagnostic tools for *M. africanum* detection, further long-term growth studies need to determine the maximum *M. africanum* - specific incubation times. Such studies could also evaluate whether the currently applied incubation protocols resulted in an underestimation of *M. africanum* prevalence or failed to detect mixed infections.

The selective advantage of the growth delay of *M. africanum* is not clear. However comparison of the genome sequences of *M. tuberculosis* H37Rv and *M. africanum* give some clues to the underlying mechanisms. First, we investigated a group of genes, each of which has already been described to result in *in vitro* growth-attenuation of *M. tuberculosis* H37Rv upon transposon (TraSH) inactivation [12]. Of 42 growth-attenuating genes, 12 genes contained non-synonymous mutations or were pseudogenes due to frameshift mutations in *M. africanum*. In particular, 4 gene products (Rv2112c/MAF_21240, Rv0862c/MAF_08710, AceE, RecA) were predicted by SIFT analysis to be affected in their protein function. These four proteins are the most likely candidates responsible for the observed growth attenuation in *M. africanum*. A fifth identified gene, *glpK*, is a pseudogene in GM041182 or with non-synonymous mutations in the other 3 strains, yet SIFT analysis identified the amino acid substitution to be tolerated by the bacteria. Therefore the Glpk protein is most likely functional in 3 out of 4 *M. africanum* strains and is not a common cause for the observed, slower growth.

We further hypothesized that a reduction and/or deficiency of molecular membrane transporters could limit growth of *M. africanum*. For instance, the knock-out of outer membrane Msp porins and a subsequent reduced sugar and phosphate uptake led to a slower growth rate of *M. smegmatis* [22]. Similarly it was previously suggested that the slower growth of *M. tuberculosis*, when compared to the fast-growing *Mycobacterium smegmatis*, could be due to the loss of several sugar transporters [17]. Therefore we aimed to determine the status of known transport mechanisms in the sequenced *M. africanum* genomes and *M. tuberculosis* H37Rv.

We identified 132 membrane transporter genes common to the two mycobacterial lineages. In *M. africanum*, there were significantly more genes encoding sulphur/sulphate-, lipid/fatty-acid, and ion-transporter with non-synonymous or frameshift mutation than in essential genes.

Although sulphur is an essential nutrient for mycobacterial survival and virulence (for review see [16], [23]), we identified (protein function affecting, SIFT) mutations in the *cysTWA/subI* ABC-transporter of *M. africanum*. Since *subI*-knock out mutants of *M. bovis* were restricted in their sulphate uptake [24], it is conceivable that *M. africanum* strains are similarly impaired in their import of sulphur. Although there was speculation that Rv1739c, another predicted sulphate transporter [25] could compensate for the loss of the *cysTWA/subI* transport system [26], it is unlikely because this protein is likewise potentially inhibited in its protein function due to a SNP mutation. Whether the hypothetical sulphate-transporter Rv1707, which carries a tolerated amino acid change in *M. africanum*, is a functional sulphate transport mechanism still has to be experimentally confirmed.

A second group of highly mutated *M. africanum* genes encode for ion transport mechanisms. Unfortunately, the knowledge about this important group of proteins is still scarce [14]. However, KefB, a potassium/proton antiporter that controls the early acidification of the phagosome, was mutated and impaired in its protein function (SIFT) in *M. africanum* [27]. Also, knock-out mutants of the Mg^2+^-transporter MgtC, which is potentially affected in its protein function (SIFT) in *M. africanum*, had impaired growth under certain *in vitro* conditions [28]. Another group of heavy-metal ion transporter genes in *M. tuberculosis*, *ctpA-ctpV*, are very different in *M. africanum*. For instance *ctpV*, one of the best studied members of this family, yet with a tolerated (SIFT) mutation in *M. africanum*, is key for mycobacterial copper homeostasis and virulence [29]. Similarly, the iron-specific ABC-transporter IrtAB, in which the IrtA subunit has an intolerable amino acid substitution (SIFT) in *M. africanum*, is not only crucial for survival in iron-deficient conditions, but is also required to effectively establish infection in the experimental murine host [30]. Most importantly, all the above mentioned ion transporter genes have one thing in common: they were found to play key roles in the intracellular survival of the bacteria within the phagolysosome of macrophages [27]–[30]. It is surprising that genes important for this crucial step of mycobacterial pathogenesis were among the least conserved in *M. africanum*, which could indicate that *M. africanum* might pursue a different intracellular survival strategy than *M. tuberculosis* to cope with the harsh environment within a phagolysosome.

Therefore we investigated 7 putative operons that were previously described as essential for the intracellular survival of *M. tuberculosis* H37Rv in macrophages [13]. Components of the sugar transport system (*sugA/B/C/lpqY*) were hyperconserved among the *M. africanum* isolates. Similarly, with the exception of *pstA1*, all *M. africanum* genes encoding phosphorous transporters were conserved amongst the sequenced isolates. Our results suggest that both pathways are equally important for *M. tuberculosis* and *M. africanum*. However, we found a remarkable difference between the two lineages. In the course of host macrophage infection, lipids become increasingly more important and replace carbohydrates as the major carbon source [17], and 3 operons of lipid metabolism (*Rv3540c*–*Rv3545c*, *Rv3550–Rv3552*, *Rv3569c–Rv3570c*) were described to be required for mycobacterial survival [13]. Interestingly, all of these operons have at least one mutated gene in *M. africanum*. Consistently, 8/14 *mmpL* genes, that likely transport lipids/fatty acids across the membrane, have altered amino acid sequences, including a frameshift mutation in *mmpL13a* that results in a pseudogene. Of note, *mmpL3*, which is one of 5 conserved *mmpL* genes in the obligate intracellular *M. leprae*, was shown to be the only gene of this family to be essential for viability of *M. tuberculosis* [31]. However, *mmpl3* is potentially affected in its protein function (SIFT analysis) in *M. africanum*. Other genes, essential for survival in macrophages, belong to a putative operon spanning from *Rv3864* to *Rv3878* [13], a genetic region that partially includes the RD1 locus encoding the ESX-1 secretion system and its virulence genes such as *esxA* (encoding ESAT-6) and *esxB* (encoding CFP-10). One of the genes, *Rv3864* (*espE*), had a non-synonymous SNP in one *M. africanum* isolate and a frameshift mutation in the remaining three sequenced *M. africanum* strains. This is interesting as *Rv3864* was associated with virulence, yet it is assumed that a loss of the gene can be compensated by its homologue *Rv3616* (*espA*) [32]. However, as the *Rv3616* (*espA*) homologue has a non-synonymous mutation in *M. africanum* as well (data not shown), it is possible that none of these genes is functional in *M. africanum*. This is supported by the previous finding that certain *M. africanum* isolates were less likely to induce an ESAT-6 dependent IFN-γ host response and it was speculated that this was due to an ESAT-6 secretion impairment [6]. Combining the finding that the *Rv3864/espE* homologue in *M. marinum* is required for secretion of CFP-10 [32], and CFP-10 contains the secretion signal of the ESAT-6/CFP-10 dimer [33], an inactive *Rv3864/espE* could therefore be the missing genetic link to explain the reduced ESAT-6 secretion of *M. africanum*. Finally, the seventh operon under study, *Rv0169–Rv0178*, is essential for entry into the mammalian cell and intracellular survival, yet several members are highly mutated in *M. africanum*. Interestingly, the overall regulator of this operon, *Rv0165* (*mce1R*), has a frameshift mutation in *M. africanum* (data not shown) and recent studies suggest that Mce1R is part of a global genome-wide regulatory network which control cell growth [34].

In the present study we found that *M. africanum* strains are impaired in their capacity to grow. We identified several potential gene candidates and functional protein groups that might contribute to the observed growth defect. To unambiguously confirm causality, complementation experiments in which the *M. africanum* mutant genes are replaced by wildtype H37Rv genes will have to be conducted. However, our genomic analysis suggests that the underlying genetic reason for the growth defect is rather complex. The growth attenuation might be a redundant result due to the loss of multiple genes. Moreover, large scale genomic analyses on additional *M, africanum* genomes will have to be conducted to confirm which of the described SNPs are really specific to all members of the *M. africanum* West Africa 2 lineage.

Taken together, from a genetic and phenotypic point of view *M. africanum* appears to be distinct from *M. tuberculosis*. *M. africanum* may have a modified, yet unknown, survival strategy of the bacterium within the human host. Future research on the lifestyle of *M. africanum* may lead to an improved understanding of growth promoting factors in *M. tuberculosis* and may ultimately reveal new strategies to interrupt bacterial growth and replication within the human host.
