# Correction: Deciphering the Growth Behaviour of *Mycobacterium africanum*

**DOI:** 10.1371/annotation/fb002e1b-e345-4832-a793-d2f4988de308

**Published:** 2013-06-11

**Authors:** Florian Gehre, Jacob Otu, Kathryn DeRiemer, Paola Florez de Sessions, Martin L. Hibberd, Wim Mulders, Tumani Corrah, Bouke C. de Jong, Martin Antonio

The figure images for Figures 2 and 3 are reversed and should be swapped: Figure 2 should replace Figure 3, and vice versa.

